# Deriving Convergent and Divergent Metabolomic Correlates of Pulmonary Arterial Hypertension

**DOI:** 10.3390/metabo13070802

**Published:** 2023-06-28

**Authors:** Mona Alotaibi, Yunxian Liu, Gino A. Magalang, Alan C. Kwan, Joseph E. Ebinger, William C. Nichols, Michael W. Pauciulo, Mohit Jain, Susan Cheng

**Affiliations:** 1 Division of Pulmonary, Critical Care and Sleep Medicine, University of California San Diego, La Jolla, CA 92093, USA; 2 Department of Cardiology, Smidt Heart Institute, Cedars-Sinai Medical Center, Los Angeles, CA 90048, USA; 3 Department of Pediatrics, College of Medicine, University of Cincinnati, Cincinnati, OH 45267, USA; 4 Division of Human Genetics, Cincinnati Children’s Hospital Medical Center, Cincinnati, OH 45229, USA; 5 Department of Medicine, University of California San Diego, La Jolla, CA 92093, USA; mjain@ucsd.edu

**Keywords:** metabolomics, convergent and divergent markers, PAH, statistical approaches

## Abstract

High-dimensional metabolomics analyses may identify convergent and divergent markers, potentially representing aligned or orthogonal disease pathways that underly conditions such as pulmonary arterial hypertension (PAH). Using a comprehensive PAH metabolomics dataset, we applied six different conventional and statistical learning techniques to identify analytes associated with key outcomes and compared the results. We found that certain conventional techniques, such as Bonferroni/FDR correction, prioritized metabolites that tended to be highly intercorrelated. Statistical learning techniques generally agreed with conventional techniques on the top-ranked metabolites, but were also more inclusive of different metabolite groups. In particular, conventional methods prioritized sterol and oxylipin metabolites in relation to idiopathic versus non-idiopathic PAH, whereas statistical learning methods tended to prioritize eicosanoid, bile acid, fatty acid, and fatty acyl ester metabolites. Our findings demonstrate how conventional and statistical learning techniques can offer both concordant or discordant results. In the case of a rare yet morbid condition, such as PAH, convergent metabolites may reflect common pathways to shared disease outcomes whereas divergent metabolites could signal either distinct etiologic mechanisms, different sub-phenotypes, or varying stages of disease progression. Notwithstanding the need to investigate the mechanisms underlying the observed results, our main findings suggest that a multi-method approach to statistical analyses of high-dimensional human metabolomics datasets could effectively broaden the scientific yield from a given study design.

## 1. Introduction

Pulmonary arterial hypertension (PAH) is a progressive disease characterized by elevated pulmonary artery pressure and, if left untreated, eventual right ventricular failure and death [1]. Despite advances in therapeutic interventions, PAH morbidity and mortality remains excessively high [2,3] and there remains a pressing need for reliable biomarkers and novel pathways to better understand the disease pathobiology. Given that metabolites can be influenced by genetics, environmental factors, and subtle disease processes, metabolomics represents a high-yield tool for identifying novel biomarkers in PAH, a complex disease that encompasses multiple types and subtypes of pathophysiologic origin and clinical presentation. Accordingly, several metabolomics investigations of PAH have emerged and already offer valuable insights regarding potentially new pathway markers that could aid in diagnosis and prognosis [4,5,6,7,8,9,10]. However, a prominent and persistent challenge for metabolomics studies of PAH, similar to that for other clinical traits, is the selection of a statistical method applied to identify associations between metabolites and disease outcome.

Because PAH is a relatively rare disease, clinical datasets tend to be smaller in sample size compared to those for other traits. Thus, the high-dimensional nature of metabolomics profiling applied in PAH studies presents a compounded challenge when considering that conventional statistical methods need to account for potentially multiple confounding factors including other metabolites, while also accounting for multiple comparisons [11,12,13,14,15]. Nonetheless, metabolomics studies of PAH have predominantly applied conventional statistical approaches such as linear regression with the false discovery rate (FDR) and Bonferroni correction for multiple tests [8,9,16]. While helpful, these methods may fail to adequately account for intercorrelations between individual metabolites, which can bias towards identifying metabolites from singular pathways and underrepresent secondary or tertiary associations from orthogonal pathways. For this reason, several alternative statistical analysis methods have been proposed and applied in other omics studies to more efficiently select features, including metabolites, associated with a given clinical trait or outcome. Therefore, we sought to formally examine different conventional and statistical learning approaches applied to metabolomics data collected from a large cohort of PAH patients. We hypothesized that the application of multiple statistical and statistical learning techniques to a high-dimensional metabolomics dataset will reveal differential rankings of convergent and divergent metabolite markers associated with our clinical outcome, PAH type. We aimed to assess the variability in findings from applying different statistical methods and the potential clinical and biological plausibility corresponding to their generated results.

## 2. Materials and Methods

### 2.1. Human Metabolomics Data and Analysis

**Figure 1:**
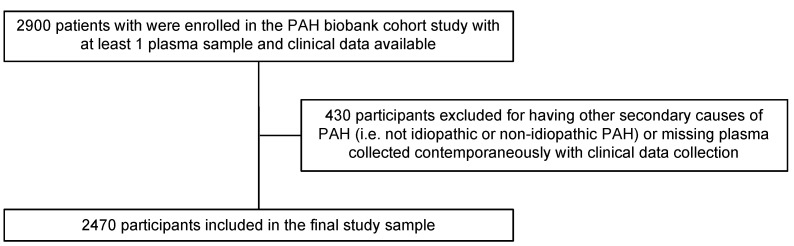
Sampling strategy.

The primary study objective was to compare results from applying different conventional statistical and statistical learning techniques to analyses of high-dimensional metabolomics data in relation to a clinical outcome. We used a cross-sectional study design to identify metabolites associated with clinically important PAH phenotypes, specifically, idiopathic PAH (IPAH) versus other types of PAH (non IPAH). We obtained patient samples from the National Biological Sample and Data Repository for PAH (PAH Biobank), an NIH-funded (R24HL105333, R01HL160941) biorepository of PAH patients enrolled from 37 U.S. centers with deidentified clinical data, stored biological samples, and genetic data. Between 2012 and 2017, the biobank enrolled 2900 PAH patients who had their plasma collected, processed, and stored at baseline. Diagnosis of PAH and PAH subtype were confirmed by right heart catheterization according to established criteria. Patients with other secondary causes of pulmonary hypertension were excluded along with patients without available plasma samples matched to clinical traits, resulting in 2470 patients remaining for the current analyses (Figure 1).

All plasma samples were collected from study patients and processed as previously described. Briefly, plasma samples were thawed at 4 °C over 8 h in light-free conditions. After thawing, the plasma was mixed thoroughly by orbital shaking at 500 rpm and 4 °C for 15 min. Cold ethanol (−20 °C) with deuterated internal standards was added to precipitate proteins, non-polar lipids, and extract bioactive lipids from the plasma. Samples were then separated using solid phase extraction (SPE) Phenomenex 8B-S199-UB) [17,18].

For profiling all plasma samples, metabolomics analyses were performed as previously described [10,16,17,18,19]. Briefly, chromatographic separation was performed on a Vanquish UPLC BEH shield RP18 column (2.1 × 100 mm, 1.7 μm) coupled to a high-resolution, QExactive orbitrap mass spectrometer (Thermo, Waltham, MA, USA) with (-) ESI. Elution was performed with mobile phase phases A (70% water, 30% acetonitrile and 0.1% acetic acid) and B (50% acetonitrile, 50% isopropanol, 0.02% acetic acid). Data were subsequently normalized to account for plate-to-plate variation using a simple batch median normalization metric with correction for median absolute deviation. Normalized, aligned, filtered datasets were subsequently used for statistical analyses. These approaches have been optimized for robust throughput, allowing for rapid extraction and analysis of thousands of plasma samples, with precision and accuracy of analytes measures found to be <20% (CV%) for 95% analytes and <20% for 87% analytes (RE%). The assay is found to be highly reproducible over long periods of time; independent measures demonstrate a median CV across analytes of 9% (range 4–27%) at low standard concentrations of 0.15 ng. All generated spectral data underwent Qc/Qa using a panel of deuterated internal standards as well as interval pooled plasma samples to monitor fluctuations in extraction efficiency, instrument sensitivity, matrix artifact, and mass accuracy. Any samples not meeting Qc/Qa thresholds underwent reinjection. Metabolites analytes were log transformed, batch normalized, and scaled.

### 2.2. Statistical Analysis

We employed a variety of statistical methods for comparison of metabolites prioritized and selected in relation to the dichotomous outcome of IPAH versus non-IPAH, given their etiologic differences (i.e., divergence) in the setting of many phenotypic similarities (i.e., convergence). All statistical analyses were conducted using R (v4.2.0), R Foundation for Statistical Computing, Vienna, Austria. URL https://www.R-project.org (accessed on 1 May 2023).

#### 2.2.1. Statistical Methods

We selected the following statistical methods: (1) univariate analyses with multiple testing correcting; (2) the least absolute shrinkage and selection operator (LASSO) approach [20]; (3) the elastic net method [21]; (4) random forest [22] with tree minimal depth variable selection [23]; (5) shrinkage discriminant analysis (SDA) [24,25]; and (6) extreme gradient boosting (XGBoost) [26]. These methods were selected based on their representing a range of conventional as well as statistical learning methods, in addition to their feasibility for application in standard research settings. Each statistical method is explained below:

##### Univariate Analyses with Multiple Testing Correction

Univariate logistic regression analyses were performed for each metabolite to predict the dichotomous outcome, with adjustments made for age and sex. The significance of each metabolite was assessed using *p*-values, which were adjusted for multiple testing. Two methods were employed for adjustment: the Bonferroni correction [27,28] or the false discovery rate (FDR) correction method [29]. The Bonferroni correction controls the family-wise error rate by dividing the desired significance level by the number of comparisons being performed, thereby reducing the chances of making a Type I error. The FDR method controls the expected proportion of false positives among all significant results. It allows for a higher number of false positives while still controlling the overall rate of false discoveries. The Bonferroni correction is more conservative than the FDR correction method as it maintains a lower overall false positive rate and, in doing so, may have reduced power to detect true effects due to its stringent adjustment. Adjusted *p*-values were calculated for each metabolite, and the metabolites were ranked based on their adjusted *p*-values, with lower values indicating higher rankings. In cases where the adjusted *p*-values were equal, ranking was performed based on the raw *p*-values.

##### Least Absolute Shrinkage and Selection Operator (LASSO)

The LASSO approach [20] was employed to perform logistic regression with variable selection. This approach incorporates a penalty term in the regression model to promote sparsity by shrinking the coefficients of less important predictors towards zero, thus retaining the most significant variables in the final model. The biglasso package [30] in R was used for LASSO logistic regression. To determine the optimal level of regularization, 10-fold cross-validation was employed, selecting the Lambda value that minimized the cross-validation error. Metabolites were ranked based on the absolute magnitude of the coefficients, with larger coefficients indicating higher ranks.

##### Elastic Net

The elastic net method [21] combines the advantage of ridge regression [31] and LASSO regression. Ridge regression assigns small but non-zero coefficients to all variables, while LASSO regression sets some coefficients exactly to zero. The elastic net method strikes a balance between feature selection and coefficient shrinkage, making it highly effective in scenarios involving correlated predictors and the need for automatic feature selection. The biglasso package [30] in R was used to conduct ridge logistic regression. To determine the optimal level of regularization, 10-fold cross-validation was utilized, selecting the Alpha and Lambda values that minimized the cross-validation error. Metabolites were ranked based on the absolute magnitude of their coefficients, assigning higher ranks to larger coefficients.

##### Random Forest

The random forest algorithm [22] was employed as a machine learning technique for prediction and feature selection. It constructs multiple decision trees by randomly sampling subsets of the data and features, combining their predictions to achieve robust and accurate results. The randomForestSRC package [32] in R was utilized for the analysis. Optimal tuning parameters, “mtry” (number of randomly selected variables) and “nodesize” (minimal size of terminal node), were determined using out-of-sample error estimation. Random forest variable selection with the tree minimal depth methodology [23,33] was then implemented. Metabolite importance was ranked using the metric proposed by Ishwaran [34] which quantifies the contribution of a particular metabolite in splitting the tree node and measures its impact on improving the accuracy of the model.

##### Shrinkage Discriminant Analysis (SDA)

Linear discriminant analysis (LDA) [35] and shrinkage linear discriminant analysis (SLDA) [24,25] were employed for classification and dimensionality reduction. SLDA addresses challenges associated with high-dimensional data and limited sample sizes by employing shrinkage techniques to enhance the estimation of covariance matrices, thereby improving classification performance. The SDA package [25,36] in R was used for SLDA. Metabolite ranking was determined by computing correlation-adjusted t (CAT) scores between the group centroids and the pooled mean [25,36,37].

##### Extreme Gradient Boosting

Extreme Gradient Boosting (XGBoost) [26] is a gradient boosting method that constructs an ensemble of weak learners, typically decision trees, in a sequential manner to minimize a specific loss function. It integrates regularization techniques to prevent overfitting and employs an advanced optimization algorithm to enhance the training process. The XGBoost package [26] in R was used for the analysis. Optimal parameters were determined using 5-fold cross-validation to minimize the binary classification error. The optimized parameters were then applied using XGBoost with the logistic regression for binary classification objective option. Metabolite importance was calculated based on the fractional contribution to the model, measured by the total gain of the metabolite’s splits.

#### 2.2.2. Comparing Performance across Statistical Methods

To compare the performance and selection of metabolites across statistical methods, we first ranked the importance of metabolites based on metrics provided by the method used: (1) the multiple testing correction method ranks metabolites based on the *p*-values obtained, with lower *p*-values corresponding to higher rankings; (2) LASSO and elastic net regression ranked metabolites based on the absolute magnitude of the coefficients, with large coefficients indicating higher ranking; (3) random forest modeling ranked metabolite importance using a metric proposed by Ishwaran [23], which quantifies how much the accuracy of the model increases due to a particular metabolite’s contribution in splitting the tree node; (4) SDA ranked metabolites by computing correlation-adjusted t (CAT) scores between the group centroids and the pooled mean [25,36,37]; and (5) XGBoost ranked the metabolites by calculating the fractional contribution of each metabolite to the model based on the total gain of the metabolite’s splits, where a higher percentage denotes more importance [26].

##### Data Structure of Differentially Prioritized Metabolites

To examine the data structures of metabolites prioritized by each of the applied statistical methods, we used Pearson correlation to quantify the inter-relations between each of the top 50 selected metabolites selected by each statistical method. A heatmap displaying the correlation structure of the selected metabolites was generated for each of the statistical methods.

##### Convergence and Divergence of Differentially Prioritized Metabolites

Individual rankings obtained from each method were summarized using several integrated rank metrics. Missing rankings were imputed for the total number of metabolites (N = 54,788). The integrated rank was calculated as the summation of rankings from each method, providing a comprehensive measure of metabolite importance. The difference rank captured the maximum difference between the highest and lowest rank for each metabolite, thus highlighting the most distinct differences in prioritization observed among the methods used. Similarly, the variance rank measured the variation in rankings across the different methods, allowing metabolites to be ranked based on the level of disagreement among the methods. These calculated rank metrics served as indicators for comparing output from across all methods applied to prioritize metabolite importance; in turn, the metrics were used to identify metabolites similarly prioritized across methods (i.e., indicating convergence) and differentially prioritized across methods (i.e., indicating divergence). 

## 3. Results

### 3.1. Cohort Characteristics

A total of 2470 patients had confirmed diagnosis of PAH (1077 IPAH and 1411 non-IPAH) and had plasma samples available for analysis and were enrolled for analysis (Table 1). The mean age of both groups was similar, with IPAH patients having a mean age of 52.16 (SD 17.83) years and non-IPAH patients having a mean age of 52.08 (SD 17.98) years (*p* = 0.9). The majority of patients in both groups were female (77.0% of IPAH patients and 78.3% of non-IPAH patients, *p* = 0.5). The mean BMI of IPAH patients was significantly higher than that of non-IPAH patients, with IPAH patients having a mean BMI of 30.44 (SD 17.63) kg/m^2^ and non-IPAH patients having a mean BMI of 28.19 (SD 11.73) kg/m^2^ (*p* < 0.001). The racial distribution of both groups was similar, with the majority of patients in both groups being white (81.4% of IPAH patients and 80.4% of non-IPAH patients). The percentages of Black, Asian, and other races were similar between both groups (*p* = 0.7).

### 3.2. Statistical Analyses of Metabolomics Data

We compared six statistical approaches including Bonferroni correction, false discovery rate (FDR) correction, LASSO, elastic net, random forest minimal depth, shrinkage discriminant analysis (SDA), and XGBoost. We selected these statistical approaches based on their complementary features. In particular, LASSO and elastic net are effective for feature selection when the number of predictors is much larger than the sample size. Bonferroni and FDR are conventionally used to correct for multiple comparisons, even though they may be overly conservative and lead to a loss of power. Random forest and XGBoost are powerful machine learning methods that can handle high-dimensional data and have been successfully applied in metabolomics previously. SDA is a method that can handle collinearity among predictors and has been applied in metabolomics for feature selection and classification tasks.

**Figure 2:**
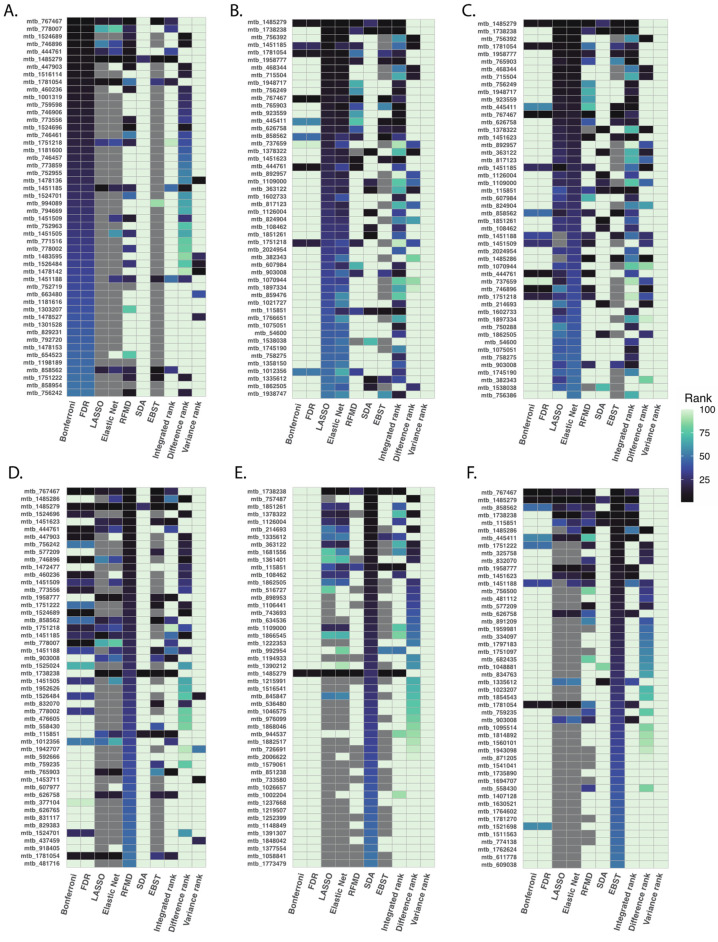
Top 50 ranked metabolites using different statistical methods. After applying six pre-specified statistical methods to our PAH dataset, we ranked metabolites based on lowest to highest *p*-values for multiple testing correction (Panel (**A**)), absolute magnitude of coefficients for LASSO and elastic net regression (Panel (**B**,**C**)), model accuracy for random forest (Panel (**D**)), correlation-adjusted t scores for SDA (Panel (**E**)), and, fractional model contribution for XGBoost (Panel (**F**)). In general, statistical approaches of similar design produced similar ranking of metabolites.

After applying the six different methods to our PAH dataset, we ranked metabolites based on measuring method-specific metrics: based on *p*-values for multiple testing correction; based on absolute magnitude of the coefficients for LASSO and elastic net regression; based on correlation-adjusted t scores for SDA; based on fractional model contribution of each metabolite for XGBoost; and based on model accuracy for random forest. In general, similar statistical approaches produced similar ranking of metabolites (Figure 2). For instance, Bonferroni correction and FDR correction produced similar ranking of metabolites, LASSO and elastic net shared similar metabolite ranking, while SDA and random forest produced significantly different ranking.

### 3.3. Correlation of Metabolites Selected by Different Statistical Methods

**Figure 3:**
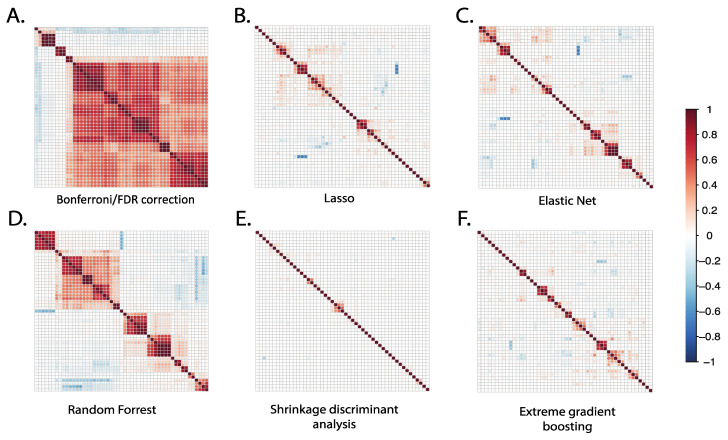
Correlation plots of the top 50 metabolites selected using different statistical methods. For each statistical method applied, we identified the 50 top-ranked metabolites selected by each method and plotted correlation matrices to examine the extent of their intercorrelations. For conventional Bonferroni/FDR methods, the top-ranked 50 metabolites demonstrated a relatively high number of intercorrelations; for other methods, the number of intercorrelations was relatively low.

The correlation between the top 50 selected metabolites from each statistical method was compared using Pearson correlation, as shown in Figure 3. The Bonferroni/FDR correction and random forest methods showed the highest correlation among the top selected metabolites, suggesting that the selected metabolites originate from a similar chemical class with chemical interdependence or homogenous representation. Accordingly, when we examined putative identities of the top ranked metabolites prioritized by these methods, they represented predominantly the following metabolite pathways: sterol and oxylipins metabolism. The results from Bonferroni and FDR correction methods showed that 82% of the top 50 metabolites were highly correlated, with correlation coefficients (R) ranging from 0.4 to 1. Similarly, the random forest method showed that more than 50% of the top 50 selected metabolites were highly correlated, with R values ranging from 0.3 to 0.9.

In contrast to results from the more conventional linear regression and random forest methods, the SDA, LASSO, XGBoost, and elastic net methods produced the lowest metabolite correlations among the top selected metabolites. The results from SDA showed that 96% of the top 50 metabolites were non-correlated, indicating different chemical pathways and broad chemical representation. This suggests that these methods may be better suited for identifying diverse metabolites that originate from different chemical classes or metabolic pathways. Indeed, when we examined putative identities of the top ranked metabolites prioritized by these methods, they represented the following metabolite pathways: eicosanoid, bile acid, fatty acid, and fatty acyl ester metabolisms. Overall, these results suggest that Bonferroni, FDR, and random forest methods produce convergent metabolite profiles, while SDA, LASSO, XGBoost and elastic net produce more divergent metabolites.

### 3.4. Metabolite Rankings across All Statistical Methods

**Figure 4:**
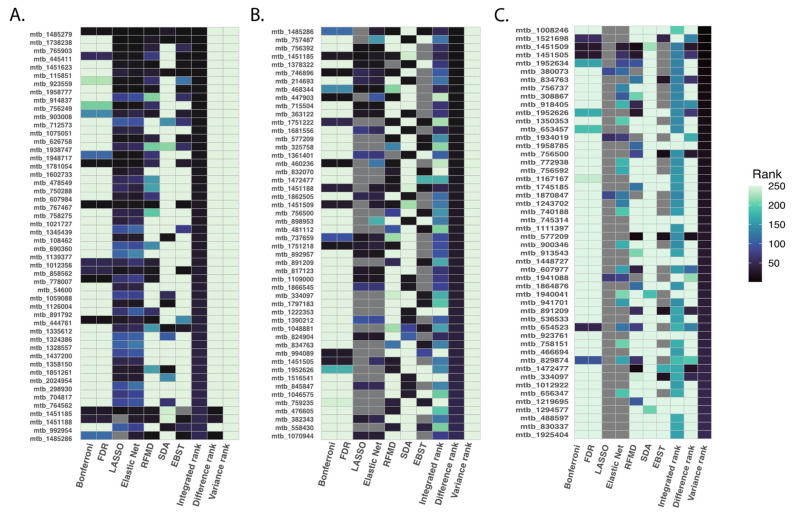
Top 50 ranked metabolites based on summarization rank metrics. To compare the priority ranks generated by all six statistical methods studied, we employed the following metrics: integrated rank was calculated as the summation of rankings from each method, difference rank was calculated as the difference between the maximum and minimum rank for each metabolite and variance rank was calculated as the variance of ranks across different methods. We focused on the top 500 metabolites using the integrated rank. The figure illustrates the top 50 ranked metabolites based on the integrated rank (Panel (**A**)), difference rank (Panel (**B**)), and variance rank (Panel (**C**)).

We applied primarily the integrated ranking method to examine how all statistical methods compared in identifying metabolites that could be considered as meeting a global ranking threshold; although each ranking method produced different sets of top metabolites, reflected by results of the difference and integrated ranking methods, the different applied methods agreed on the most highly prioritized analytes (Figure 4).

## 4. Discussion

Metabolomics and complementary omics investigations typically involve analyzing high-dimensional data structures that include a large and continually expanding number of variable measures. Identifying the variable measures from these datasets that carry potentially the greatest clinical relevance, in relation to meaningful outcomes, is analytically challenging and requires rigorous statistical methods. For metabolomics, in particular, technical advances in mass spectrometry methods continue to offer increasing sensitivity for detecting small molecule analytes such that the total number of analytes that can be profiled from a single experimental run is exponentially larger than what was possible just over a decade ago. The ability to detect and measure a growing number of total analytes has, in turn, augmented the challenge of statistically analyzing datasets containing a growing number of measures that are likely to demonstrate at least some degree of intercorrelation as well as interactions in relation to a potential outcome of interest.

In the present study, we sought to clarify the statistical analytical issues relevant to contemporary human metabolomics studies. In particular, we compared different conventional statistical and machine learning (i.e., statistical learning) approaches to identifying metabolites associated with the PAH disease type in a large cohort of patients for whom comprehensive high-dimensional metabolomics profiling was conducted. Specifically, we compared six different statistical approaches that can be used for variable selection: Bonferroni/FDR correction, LASSO, elastic net, random forest, SDA, and XGBoost. We showed that similarly constructed statistical approaches produced similar rankings of metabolites. Bonferroni/FDR correction and random forest demonstrated the highest correlated results for top-ranked metabolites, while SDA and random forest produced least correlation among the top-ranked metabolites. These differences in results corresponded to putative metabolite pathways with biologically plausible complementary and potentially orthogonal relations to PAH pathophysiology.

Metabolomics studies have emerged as an important approach to investigating metabolic dysregulations in complex clinical disease conditions such as PAH. Several metabolomics studies conducted in patients with PAH, in particular, have identified numerous metabolites associated with the disease, including analytes that reflect alterations in amino acid metabolism and lipid peroxidation [4,8,9,10,16]. Most studies in PAH metabolomics have employed conventional statistical approaches such as univariate and multivariate analyses and utilized two common methods for multiple test correction: Bonferroni and FDR. Although these statistically conservative approaches can be useful for targeted metabolomics studies with a small number of metabolites, they may offer limited sensitivity for detecting associations among high-dimensional data, which are characteristic of untargeted metabolomics studies. This issue is particularly relevant to relatively rare disease phenotypes, such as PAH, for which the number of measured metabolites is likely to exceed the number of observations in a given study cohort by several-fold. To this end, approaches such as LASSO, elastic net, and other statistical learning (i.e., machine learning) algorithms offer efficient as well as informative alternatives to conventional data analysis approaches.

Beyond the efficiency of statistical learning approaches is the ability to account for intrinsic intercorrelation of metabolites in a biological system, which stands as a persistent analytical challenge for metabolomics studies. Metabolite intercorrelations may arise from a number of sources including similarities in chemical structure, function, or enzymatic derivation. Statistically, metabolite intercorrelations can result in the prioritization of metabolites from a singular biological pathway at the cost of underrecognizing other metabolites. Although this can be useful for identifying biomarker panels for disease, as well as exploring putative primary underlying mechanisms and networks, pathway singularity may preclude discovery of potentially orthogonal disease markers or therapeutic targets. Intercorrelations also pose challenges for statistical interpretation, as they may result in multicollinearity, overfitting, and inflated false positive rates. Accordingly, our study showed that statistical learning methods such as SDA and random forest, as an alternative to conventional statistical approaches, can produce substantially different rankings of relatively diverse metabolites. Whereas conventional methods tended to prioritize sterol and oxylipin metabolites in relation PAH traits, the statistical learning methods tended to prioritize eicosanoid, bile acid, fatty acid, and fatty acyl ester metabolites. Each of these pathways represents highly biological plausible mechanistic contributors to the PAH disease type and pathogenesis. While the current study is focused on delineating statistical approaches that can be used to identify such convergent and divergent results, additional work is needed to further investigate the mechanisms underlying these observations. For PAH studies, in particular, next steps can include validating the findings in separate cohorts enriched with similar clinical outcomes and then examining the extent to which convergent metabolites (i.e., sterol and oxylipin analytes) may demonstrate concordant pathway activity in basic and translational models. Importantly, if also validated in separate clinical as well as mechanistic studies, the apparently divergent metabolites (i.e., eicosanoid, bile acid, fatty acid, and fatty acyl ester analytes) may be found to represent pathways that could be therapeutically targeted to achieve outcomes specific to a particular PAH subtype, patient subgroup, or severity level of clinical disease.

The novelty of this work stems from leveraging a large human metabolomics dataset and comparing different statistical and machine learning approaches to identify metabolite associations with the same clinical outcome. This comprehensive analysis offers insights regarding the performance and consistency of various statistical methods for variable selection in metabolomics studies. Additionally, this study underscores the challenge of metabolite intercorrelations as a persistent analytical issue for metabolomics studies. We demonstrate how conventional statistical methods may tend to prioritize metabolites from singular biological pathways, potentially overlooking orthogonal disease pathway markers. By contrast, statistical learning methods such as SDA and random forest tend to prioritize more divergent and diverse metabolites. Therefore, the selection of an approach for analyzing human metabolomics data should be carefully considered based on the clinical question at hand, and it may be worthwhile to consider employing multiple approaches when conducting non-targeted discovery metabolomics studies.

Although our study does not directly translate into immediate clinical implications, it emphasizes the importance of employing a combination of different statistical methods. This approach can uncover novel and interconnected pathways that contribute to disease pathogenesis. By embracing a multi-method strategy, researchers can enhance their understanding of complex diseases and potentially identify new therapeutic targets and diagnostic markers. Therefore, our findings underscore the value of utilizing diverse statistical approaches in advancing medical research and uncovering new insights into disease mechanisms.

Several limitations of our study merit consideration. We applied a limited number of statistical methods to a single PAH dataset, albeit including data from samples collected across multiple sites. Additional work is needed to evaluate a potentially broader set of statistical approaches and future investigations may also involve validating results in separate cohorts. We predicated all modeling on analyses of a single outcome variable, rather than a range of clinical variables, for the sake of efficiency and given the key clinical importance and representative nature of this outcome. We anticipate that future work exploring additional clinical outcomes in relation to PAH or other disease traits is likely to yield similar findings with some variation depending on the clinical question and size and structure of the dataset.

## 5. Conclusions

Utilizing a comprehensive PAH metabolomics dataset, we compared six different statistical and statistical learning techniques to identify metabolites associated with PAH type. Our results revealed that conventional methods, such as Bonferroni/FDR correction, often prioritized highly inter-correlated metabolites. In contrast, statistical learning techniques showed agreement with conventional methods in ranking top metabolites while also encompassing a broader range of metabolite groups. In conclusion, our findings demonstrate that the process of identifying clinically relevant variables from high-dimensional metabolomics data requires careful consideration of the statistical methods available for analyses. The statistical approaches applied in our study were all effective for achieving variable selection and, when applied in parallel as opposed to in isolation, produced a set of both convergent and divergent novel markers. Thus, in addition to a careful selection of the primary statistical method being applied to fit a specific clinical question or analytical goal, future investigations may also consider a multi-method approach to broaden the potential information gained from a given study design.

## Figures and Tables

**Table 1:** Cohort characteristics.

Characteristic	All Patients	Idiopathic PAH	Non-Idiopathic PAH	*p*-Value *
N	2488	1077	1411	
Age, years	52.1 (17.9)	52.16 (17.83)	52.08 (17.98)	0.9
Female (%)	1934 (77.7)	829 (77.0)	1105 (78.3)	0.4
BMI, kg/m^2^	29.16 (14.61)	30.44 (17.63)	28.19 (11.73)	<0.001
Race (%)				0.7
White	2011 (80.8)	877 (81.4)	1134 (80.4)	
Black	309 (12.4)	131 (12.2)	178 (12.6)	
Asian	92 (3.7)	39 (3.6)	53 (3.8)	
Other	76 (3.1)	30 (2.7)	46 (3.3)	

Data are displayed as mean and standard deviation (SD) unless specified otherwise. * *p*-values are calculated based on comparisons between idiopathic and non-idiopathic PAH groups.

## Data Availability

The datasets generated and/or analyzed during the current study are available in the National Biological Sample and Data Repository for PAH: pahbiobank.org. Accessed 31 January 2023.
